# Prolactin-Induced Adaptation in Glucose Homeostasis in Mouse Pregnancy Is Mediated by the Pancreas and Not in the Forebrain

**DOI:** 10.3389/fendo.2021.765976

**Published:** 2021-11-12

**Authors:** Zin Khant Aung, Ilona C. Kokay, David R. Grattan, Sharon R. Ladyman

**Affiliations:** ^1^ Centre for Neuroendocrinology, Department of Anatomy, University of Otago, Dunedin, New Zealand; ^2^ Maurice Wilkins Centre for Molecular Biodiscovery, University of Auckland, Auckland, New Zealand

**Keywords:** prolactin, pregnancy, beta-cell, hypothalamus, prolactin receptor, gestation

## Abstract

Adaptive changes in glucose homeostasis during pregnancy require proliferation of insulin-secreting beta-cells in the pancreas, together with increased sensitivity for glucose-stimulated insulin secretion. Increased concentrations of maternal prolactin/placental lactogen contribute to these changes, but the site of action remains uncertain. Use of Cre-lox technology has generated pancreas-specific prolactin receptor (Prlr) knockouts that demonstrate the development of a gestational diabetic like state. However, many Cre-lines for the pancreas also express Cre in the hypothalamus and prolactin could act centrally to modulate glucose homeostasis. The aim of the current study was to examine the relative contribution of prolactin action in the pancreas and brain to these pregnancy-induced adaptations in glucose regulation. Deletion of prolactin receptor (Prlr) from the pancreas using Pdx-cre or Rip-cre led to impaired glucose tolerance and increased non-fasting blood glucose levels during pregnancy. Prlr^l^ *^ox/lox^*/Pdx-Cre mice also had impaired glucose-stimulated insulin secretion and attenuated pregnancy-induced increase in beta-cell fraction. Varying degrees of Prlr recombination in the hypothalamus with these Cre lines left open the possibility that central actions of prolactin could contribute to the pregnancy-induced changes in glucose homeostasis. Targeted deletion of Prlr specifically from the forebrain, including areas of expression induced by Pdx-Cre and Rip-cre, had no effect on pregnancy-induced adaptations in glucose homeostasis. These data emphasize the pancreas as the direct target of prolactin/placental lactogen action in driving adaptive changes in glucose homeostasis during pregnancy.

## Introduction

During pregnancy, glucose regulation undergoes major adaptations to ensure a consistent supply of glucose to the fetus (1). Glucose transport across the placenta is a passive process and requires a concentration gradient with the maternal side having higher glucose levels than the fetal side. As the fetus grows and requires more glucose, this gradient is challenged. To establish higher maternal glucose levels and thereby maintain the gradient, many maternal tissues develop a state of relative insulin resistance. Conversely, to protect the fetus from excessively high glucose levels, such as following a meal, the maternal pancreatic beta-cells develop an increased capacity to respond to acute increases in maternal blood glucose. This adaptive response involves proliferation of the insulin-secreting beta-cells in the pancreas, together with increased sensitivity of glucose-stimulated insulin secretion (2). Dysfunction of glucose regulation during pregnancy, particularly when beta-cells fail to compensate for the insulin resistance of late pregnancy, leads to gestational diabetes mellitus, which can have long term adverse consequences on the mother and offspring.

The expansion of the beta-cells occurs in anticipation of increased insulin demand, and as such is a hormone-induced adaptation to prepare for future changes in physiological requirements. The anterior pituitary hormone prolactin and its pregnancy-specific homologue placental lactogen, which also acts through the prolactin receptor (Prlr), play key roles in mediating the adaptations of the beta-cells during pregnancy (3–6). There is convincing, although largely circumstantial, evidence that prolactin and/or placental lactogen acts directly on the beta-cell to induce both proliferation and enhanced glucose-stimulated insulin secretion (GSIS). Beta-cells express Prlr and expression is increased during pregnancy (7). *In vitro* studies demonstrate that prolactin increases insulin secretion and beta-cell proliferation (6, 8), and in rats with chronically high prolactin, insulin secretion is enhanced (9). Furthermore, evidence suggests that the pregnancy-induced beta-cell adaptations require intact Prlr signaling. Prlr knockout mice cannot sustain a pregnancy (10), due to loss of prolactin action on the corpus luteum of the ovary, but heterozygous Prlr knockout mice show attenuated pregnancy-induced beta-cell expansion and impaired glucose tolerance (4). More recently, conditional deletion of Prlr from beta-cells, leaving Prlr in the ovary intact, resulted in mice that were able to sustain a pregnancy but developed gestational diabetes (11, 12). While these latter studies provide the most convincing *in vivo* evidence of direct action of prolactin in the pancreas mediating beta-cell expansion during pregnancy, a critical caveat is that both the Cre lines used to generate these transgenic mice (Rip-Cre and Pdx-Cre, respectively) have “ectopic” Cre expression in the brain and there has been no assessment in either study examining the potential contribution of deletion of Prlr in the brain.

There is extensive evidence that, in addition to the beta-cells, the Rip-Cre transgene drives recombination of floxed genes in the brain, particularly in parts of the hypothalamus involved in regulation of glucose homeostasis (13–15). Indeed, we have recently shown prolactin-induced pSTAT5 [an indicator of prolactin activation of intracellular signaling within the brain (16)] in Rip-Cre neurons and Rip-Cre-mediated deletion of Prlr in various regions of the hypothalamus and the amygdala when using Rip-Cre transgenic mice crossed with Prlr flox mice (17). The expression of Cre in the brain, particularly the hypothalamus, in pancreas- or beta-cell-specific Cre mouse lines is not limited to just the Rip-cre transgene. The extent of the problem is such that an influential report examining various genetic manipulations to target the pancreas concluded that assessment of possible deletion of target genes from the brain also needs to be considered before accurate assessment of the contribution of the pancreas to the observed phenotype can be determined (15). This has not yet been done for the role of Prlr in driving changes in beta-cells.

The aim of this study is to address this deficit, investigating the contribution of prolactin/placental lactogen action in the pancreas and the hypothalamus in the pregnancy-induced adaptations of glucose homeostasis. The hypothalamus plays a major role in metabolic homeostasis, including regulation of insulin secretion and whole-body glucose metabolism (18–20). We have demonstrated extensive expression of Prlr in regions of the hypothalamus involved in glucose homeostasis, including the arcuate, ventromedial and paraventricular nuclei of the hypothalamus (16, 21). Furthermore, many brain regions that project onto autonomic afferents of the pancreas, including medial preoptic area, anterior bed nucleus of stria terminalis, medial amygdala, paraventricular nucleus, area postrema, lateral parabrachial nucleus and periaqueductal grey matter (22–24) all contain neurons that are prolactin-responsive (25). Hence, at least part of the pregnancy-induced enhancement of insulin production might be mediated through prolactin-sensitive autonomic output from the CNS. Prlr in the brain mediate multiple adaptive responses to help the mother prepare for the demands of pregnancy and lactation (26), including leptin resistance (27, 28), increased food intake and fat deposition in anticipation of metabolic demands of lactation (27, 29); suppression of the reproductive axis and maintenance of lactational infertility (30, 31); increased neurogenesis (32), reduced physical activity (33) and establishment of maternal behavior (34). Moreover, chronic prolactin infusion into the brain can increase beta-cell mass by enhancing beta-cell proliferation in diabetic rats (35), consistent with the hypothesis that central activation of Prlr may contribute to beta-cell expansion during pregnancy.

## Methods and Materials

### Animals

*Prlr* ^lox/lox^ mice (36) and Prlr-iCre mice were generated as previously described (21, 36, 37). Prlr-iCre mice were crossed with ROSA26-CAGS-τGFP reporter mice (38), generating mice that express τGFP specifically in Prlr-expressing cells (21). For the *Prlr* ^lox/lox^ mice, in the presence of Cre-recombinase the sequence of the *Prlr* gene between the lox66 and lox71 sites undergoes inversion, resulting in the deletion of exons 5-10 of the *Prlr* gene and expression of enhanced green fluorescent protein (GFP). To generate mice with Prlr deleted from the pancreas and beta-cells, female *Prlr* ^lox/lox^ mice were crossed with male mice expressing Cre-recombinase (Cre) in *Rip* containing cells (Rip-Cre, B6.Cg-Tg(Ins2-Cre)25Mgn/J, obtained from The Jackson Laboratory #003573) or *pdx* containing cells (Pdx-Cre, B6.FVB-Tg(Pdx1-cre)6Tuv/J, obtained from The Jackson Laboratory #014647) respectively. As both of these Cre lines also express Cre in the forebrain to varying degrees, we also generated mice with Prlr deleted specifically from the forebrain by crossing female *Prlr* ^lox/lox^ mice with mice in which Cre expression was driven by the neuron-specific calcium/calmodulin dependent protein kinase II-alpha (39) (CamK-Cre).

Experiments used female mice aged 8-12 weeks old, housed in a temperature- and lighting- controlled environment (22 ± 1 C, 12 h light:12 h dark, lights on at 0800h) and allowed access to food and water *ad libitum* except during fasting when only water was available. All experimental protocols were approved by the University of Otago Animal Ethics Committee. To generate timed pregnancies, female mice were housed with a male until pregnancy was confirmed by the presence of a mucous plug in the morning following mating (day 1 of pregnancy). This study only used mice in their first pregnancy.

### Immunohistochemistry

Mice were anesthetized with sodium pentobarbital and perfused transcardially with 4% paraformaldehyde. Brains and pancreata were removed and post-fixed for one hour in 4% paraformaldehyde, then cryoprotected in 30% sucrose at 4°C overnight. For brains, coronal sections (30µm) were cut through the hypothalamus at the level of the arcuate nucleus and sections were then processed for free-floating chromogen immunohistochemistry for GFP using an anti-GFP antibody (1:20000 dilution, polyclonal rabbit-anti GFP, A6455, Thermo Fisher Scientific, MA, USA, RRID : AB_221570) as previously described (36). For the pancreas, sections (16 µm) were sliced and float mounted on slides. Immunohistochemistry for GFP was carried out as described above. The pancreas sections from pregnant mice were also used for chromogen immunohistochemistry for detection of pSTAT5 using an anti-phospho-STAT5 antibody (1:400 dilution polyclonal rabbit anti-phospho-STAT5, Tyr 694, #9351 Cell Signaling Technology, MA, USA, RRID : AB_2491009) as previously described (16). Pancreata were collected from Prlr-iCre/eR26-τGFP mice after transcardial perfusion with 4% paraformaldehyde. Tissue was post-fixed in 30% sucrose at 4°C overnight then stored at -80°C until sections were sectioned and float mounted onto slides. Sections were then processed for GFP immunohistochemistry as previously described (21).

### *In Situ* Hybridization

For *in situ* hybridization assessment of Prlr mRNA, mice were perfused with 2% paraformaldehyde and pancreata were collected and cryoprotected in 30% sucrose at 4°C overnight. Sections (16 µm) were collected through the pancreas and underwent *in situ* hybridization using an S^35^-labelled RNA probe targeted to the mRNA for the long form of the Prlr as described previously (16).

### Glucose Tolerance Tests, Glucose-Stimulated Insulin Concentrations and Non-Fasting Glucose and Insulin Concentrations

All blood glucose levels were measured at the time of blood collection using a glucometer (Accu-Chek Performa; Roche, Mannheim, Germany). Glucose tolerance tests (GTT) and glucose stimulated insulin secretion (GSIS) experiments were performed in mice after an overnight fast. For GTTs, basal blood glucose levels were measured at -30 minutes and then immediately before mice received an i.p. injection of glucose (1g/kg dissolved in saline) and then at various time points after injection. For GSIS, mice were injected with either saline or glucose (1 g/kg dissolved in saline) and were decapitated 10 minutes later. Trunk blood was collected, spun down and plasma was stored at -20°C until insulin concentration was determined using an ultra-sensitive mouse insulin ELISA (Crystal Chem, Inc., Downer’s Grove, IL, USA, RRID : AB_2783626). For non-fasting blood glucose levels, mice were rapidly decapitated and blood glucose was measured immediately in trunk blood, which was then collected, and processed as described above for determination of insulin concentration.

### Beta-Cell Fraction Analysis

Pancreata were collected for immunohistochemistry as described above and stored at -80°C until further processing. Pancreatic tissue was embedded in paraffin and cut in 4 µm thick sections. Pancreatic sections used for quantitative analysis were systematically selected after a random start and three sections (200 µm apart) per pancreas were included in analysis. To identify beta-cells, pancreatic sections underwent chromogen immunohistochemistry for insulin using an anti-insulin antibody (ab63820 Abcam, Cambridge UK RRID : AB_1925116). Gills Hematoxylin was used as a counter stain. To determine beta-cell fraction, each 4 µm section (n=3 per mouse) was imaged using an Olympus Montaging Microscope, and 10-30 non-overlapping fields of view were photographed per section. These images were adjacent to one another and covered the entire pancreatic area on the slide. The images were put together into a montage using Autopano Pro software and then analyzed using ImageJ software to determine the area of total pancreatic tissue and the area stained for insulin. Beta-cell fraction was determined by dividing total area stained for insulin by total area of pancreatic tissue in the section.

### Glucose Homeostasis During Pregnancy in Prlr^lox/lox^/Rip-Cre Mice

Pregnant female *Prlr* ^lox/lox^/Rip-Cre mice and controls (both Cre-negative/flox-positive (*Prlr* ^lox/lox^) and Cre-positive/flox-negative (Rip-Cre) mice) underwent a GTT on day 15 or day 16 of pregnancy. Non-fasting blood glucose levels were determined in another cohort of pregnant *Prlr* ^lox/lox^/Rip-Cre mice and controls. GFP immunohistochemistry was carried out on pancreas and brain sections from a third cohort of pregnant *Prlr* ^lox/lox^/Rip-Cre mice and *Prlr* ^lox/lox^ mice. Body weight (day 1 to day 18 pregnancy) and food intake (day 9 to day 18 pregnancy) was monitored in another cohort of *Prlr* ^lox/lox^/Rip-Cre mice and controls (Cre-negative/flox-positive only).

### Hypothalamic Expression of Recombination in Mice With Prlr Deletions Using Different Cre-Lines

The extent of Rip-Cre-mediated recombination in the brain was such that we decided to compare with both a forebrain-specific Cre (CamK-Cre) and a second pancreas-specific Cre (Pdx-Cre). Sections collected from the hypothalamus of pregnant and non-pregnant female control (*Prlr* ^lox/lox^), *Prlr* ^lox/lox^/Rip-Cre, *Prlr* ^lox/lox^/Pdx-Cre and *Prlr* ^lox/lox^/CamK-Cre mice underwent immunohistochemistry to detect GFP positive cells as described above. Immunohistochemistry was examined and photographed using an Olympus AX70 research light microscope (Olympus, Tokyo, Japan). GFP-positive cells in the arcuate nucleus [-1.34 to -2.30 from bregma (40)] were counted in at least 3 sections per mouse using Image J software. For each mouse, the average number of positive cells per section was calculated and used for statistical comparisons between different groups.

### Glucose Homeostasis During Pregnancy in Prlr^lox/lox^/Pdx-Cre and Prlr^lox/lox^/CamK-Cre Mice

Control (*Prlr* ^lox/lox^), *Prlr* ^lox/lox^/Pdx-Cre and *Prlr* ^lox/lox^/CamK-Cre mice underwent a GTT both prior to mating and on day 15/16 of pregnancy. A second cohort of female control (*Prlr* ^lox/lox^), *Prlr* ^lox/lox^/Pdx-Cre and *Prlr* ^lox/lox^/CamK-Cre mice were perfused either as virgin controls or on day 16 of pregnancy and pancreata were collected for immunohistochemistry for insulin. GSIS, non-fasting blood glucose levels and insulin concentrations were assessed in further cohorts of non-pregnant and pregnant *Prlr* ^lox/lox^ and *Prlr* ^lox/lox^/Pdx-Cre mice. Non-fasting plasma insulin concentration were measured in trunk blood samples from a second cohort of day 16/17 pregnant *Prlr* ^lox/lox^/CamK-Cre and control (*Prlr* ^lox/lox^) mice. The pancreata of pregnant *Prlr* ^lox/lox^ and *Prlr* ^lox/lox^/Pdx-Cre mice were collected and processed for immunohistochemistry for either GFP or pSTAT5 to characterize the deletion of Prlr in the pancreas of this transgenic mouse. Pancreas tissue was collected from another group of pregnant *Prlr* ^lox/lox^ and *Prlr* ^lox/lox^/Pdx-Cre mice for *in situ* hybridization for *Prlr* mRNA.

### Statistical Analysis

Data are presented as mean ± SEM, and all statistical analysis was undertaken using GraphPad Prism 6 (GraphPad). GFP-positive cell counts, GTT area under the curve, fasting and non-fasting glucose concentrations were analyzed by Student’s t-test or one-way ANOVA if there were more than two groups followed by *post hoc* Tukey’s multiple comparison test when necessary. Insulin concentrations and beta-cell fraction in non-pregnant and pregnant groups were analyzed by two-way ANOVA. Comparison of beta-cell fractions in the non-pregnant state was analyzed by one-way ANOVA.

## Results

**Figure 1:**
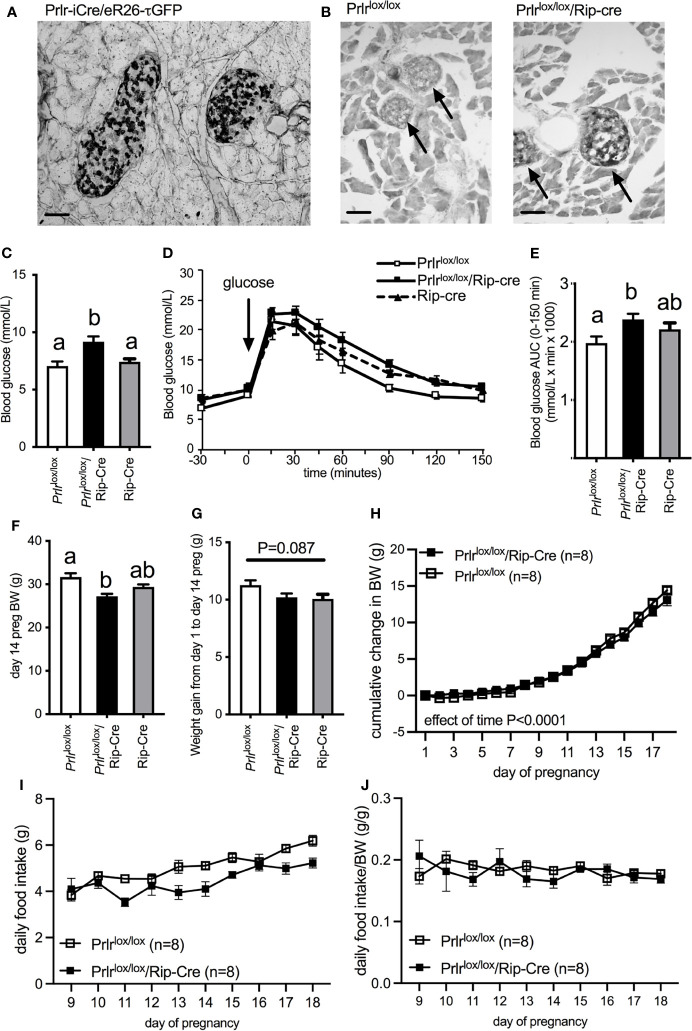
**(A)** GFP expression in the pancreas of Prlr-iCre/eR26-τGFP mice in which Prlr-expressing cells are labeled with τGFP. GFP was visualized with immunohistochemistry using DAB (indicated by black in image). GFP expression in the pancreas from Prlr-iCre/eR26-τGFP mice was extensively localized to islets. **(B)** Representative images of immunohistochemistry for GFP in the pancreas of *Prlr* ^lox/lox^ and *Prlr* ^lox/lox^/Rip-Cre mice. Expression of GFP in these mice is indicative of the recombination of the targeting construct and deletion of the *Prlr* gene, and was only observed in the islets of *Prlr* ^lox/lox^/Rip-Cre mice and not control mice. Arrows indicate position of islets within the pancreatic tissue. **(C)** Blood glucose levels on day 15/16 of pregnancy in non-fasted *Prlr* ^lox/lox^/Rip-Cre and control (Rip-cre only and *Prlr* ^lox/lox^ only) mice. Groups with different letters are significantly different. **(D)** Glucose tolerance in day 15/16 pregnant *Prlr* ^lox/lox^/Rip-Cre and control (Rip-cre only and *Prlr* ^lox/lox^ only) mice. Blood glucose was monitored before and after the administration of 1g/kg glucose in fasted mice. **(E)** Area under the curve (mean± SEM) during glucose tolerance tests. Groups with different letters are significantly different. P<0.05, n = 5-15 per group. **(F)** Body weight on day 14 (prior to fasting for GTT) for *Prlr* ^lox/lox^/Rip-Cre and control (Rip-cre only and *Prlr* ^lox/lox^ only) mice. Groups with different letters are significantly different. **(G)** Weight gain from day 1 to day 14 of pregnancy for *Prlr* ^lox/lox^/Rip-Cre and control (Rip-cre only and *Prlr* ^lox/lox^ only) mice. **(H)** Cumulative weight gain across pregnancy in *Prlr* ^lox/lox^/Rip-Cre and *Prlr* ^lox/lox^ mice. **(I)** Daily food intake from day 9 to day 18 of pregnancy in *Prlr* ^lox/lox^/Rip-Cre and *Prlr* ^lox/lox^ mice. **(J)** Daily food intake normalized to body weight from day 9 to day 18 of pregnancy in *Prlr* ^lox/lox^/Rip-Cre and *Prlr* ^lox/lox^ mice. Scale bars indicate 50µm.

Using a Prlr reporter line (Prlr-iCre X ROSA26-CAGS-τGFP), GFP staining indicative of Prlr expression was localized to islets within the pancreas (**Figure 1A**). In the conditional knockout mice using Rip-cre to target the pancreas, positive GFP staining was readily observed in the islet cells from *Prlr* ^lox/lox^/Rip-cre (**Figure 1B**) indicative of Prlr promotor driven expression of the reporter following Cre-mediated recombination of the *Prlr* gene. No positive GFP staining was observed in the pancreas from control (*Prlr* ^lox/lox^) mice (**Figure 1B**).

Pregnant *Prlr* ^lox/lox^/Rip-Cre mice had elevated non-fasting blood glucose concentrations compared to either pregnant Rip-cre or *Prlr* ^lox/lox^ mice (one-way ANOVA P=0.0036) (**Figure 1C**). Pregnant *Prlr* ^lox/lox^/Rip-Cre mice had impaired glucose tolerance compared to pregnant *Prlr* ^lox/lox^ mice, however there was no difference in glucose tolerance between pregnant *Prlr* ^lox/lox^/Rip-Cre and Rip-Cre mice, suggesting that the Cre, by itself, was having an adverse effect on glucose tolerance in pregnancy (one-way ANOVA P=0.042) (**Figures 1D, E**). Previously, we have reported that *Prlr* ^lox/lox^/Rip-Cre non-pregnant female mice have slightly impaired glucose tolerance and lower body weight compared to *Prlr* ^lox/lox^ mice and Rip-Cre mice (17). Although, day 14 pregnant *Prlr* ^lox/lox^/Rip-Cre mice (BW taken before fasting for GTT) had significantly lower body weight than Cre-negative *Prlr* ^lox/lox^ mice (controls) at the same stage of pregnancy (one-way ANOVA P=0.0013, Tukey’s multiple comparison test *Prlr* ^lox/lox^/Rip-Cre vs *Prlr* ^lox/lox^: P<0.05) (**Figure 1F**) they had similar weight gain during pregnancy as that seen in controls (one-way ANOVA P=0.087) (**Figure 1G**). In addition, in a separate cohort of mice that did not undergo a GTT during pregnancy, gestational weight gain was similar for the entire pregnancy (repeated measures ANOVA, effect of genotype P=0.6803) (**Figure 1H**). Both *Prlr* ^lox/lox^/Rip-Cre and control (*Prlr* ^lox/lox^) mice increased food intake during the second half of pregnancy (Interaction time X genotype P=0.0688, Effect of time P<0.0001) (**Figure 1I**) yet pregnant *Prlr* ^lox/lox^/Rip-Cre had lower food consumption compared to controls (Effect of genotype P=0.0029) (**Figure 1I**). Similar to our previous work in virgin *Prlr* ^lox/lox^/Rip-Cre mice (17), when food intake was normalized to body weight in pregnancy, there was no significant difference in food intake between *Prlr* ^lox/lox^/Rip-Cre and control *Prlr* ^lox/lox^ mice (Effect of genotype P=0.5516) (**Figure 1J**).

**Figure 2:**
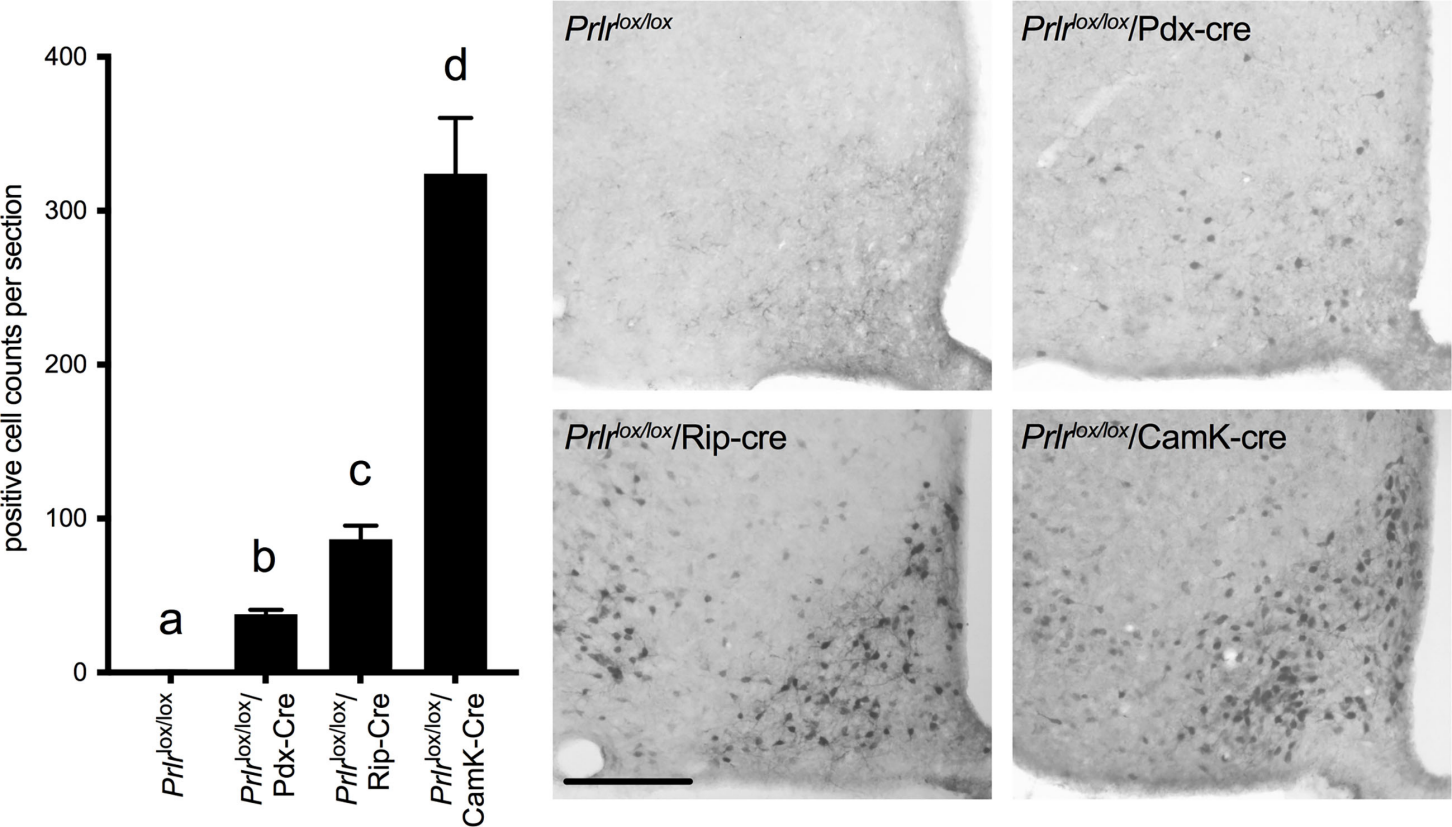
Immunohistochemistry for GFP in the arcuate nucleus of *Prlr* ^lox/lox^, *Prlr* ^lox/lox^/Pdx-Cre, *Prlr* ^lox/lox^/Rip-Cre and *Prlr* ^lox/lox^/CamK-Cre mice. Expression of GFP is indicative of the recombination of the targeting construct and deletion of the *Prlr* gene. Numbers of cells in the arcuate nucleus positively stained for GFP were quantified in *Prlr* ^lox/lox^ and mice with conditional deletion of Prlr (n = 6-8 per group). Cell count analysis was carried out in pregnant *Prlr* ^lox/lox^/Rip-Cre, *Prlr* ^lox/lox^/CamK-Cre and control mice, while *Prlr* ^lox/lox^/Pdx-Cre and controls were a mixture of day 15 pregnant and non-pregnant mice (as there was no difference between pregnant and non-pregnant mice). Bars with different letters are significantly different to each other, P<0.05. Scale bars indicate 50µm.

As we have previously observed in virgin *Prlr* ^lox/lox^/Rip-Cre mice (17), there was a large number of GFP-positive cells in the arcuate nucleus of the hypothalamus (**Figure 2**) and several other brain regions (rostral preoptic area, ventrolateral region of the ventromedial nucleus of the hypothalamus, medial tuberal nucleus and the medial amygdala) indictive of *Prlr* recombination in these cells. In the *Prlr* ^lox/lox^/Pdx-Cre mice, GFP expression in the brain was limited to the arcuate nucleus, and was significantly lower than that seen in *Prlr* ^lox/lox^/Rip-Cre mice (one-way ANOVA P<0.0001) (**Figure 2**). To determine if deletion of Prlr in the brain was contributing to any effects on glucose homeostasis during pregnancy, we also included in our studies mice with a forebrain-specific deletion of the Prlr using a CamK-Cre driven promotor. *Prlr* ^lox/lox^/CamK-Cre mice showed positively-stained GFP cells throughout the hypothalamus, as previously reported (36), and had high GFP expression in the arcuate nucleus, indicative of deletion of the Prlr (**Figure 2**). Previously, we have reported attenuated prolactin-induced pSTAT5 in the arcuate nucleus of *Prlr* ^lox/lox^/CamK-Cre demonstrating greatly reduced Prlr activity indicative of Prlr deletion (33, 36, 41). Since brain GFP expression in *Prlr^lox/lox^*/Pdx-Cre was significantly less than *Prlr* ^lox/lox^/Rip-Cre mice (**Figure 2**), limited to just the arcuate nucleus, and the pregnant *Prlr* ^lox/lox^/Rip-Cre mice glucose tolerance results were compromised by effects of the Rip-Cre transgene itself (**Figures 1D, E**), only *Prlr^lox/lox^*/Pdx-Cre mice were subsequently used for experiments to determine the contribution of prolactin action in the pancreas and brain in mediating pregnancy-induced adaptations in glucose homeostasis.

**Figure 3:**
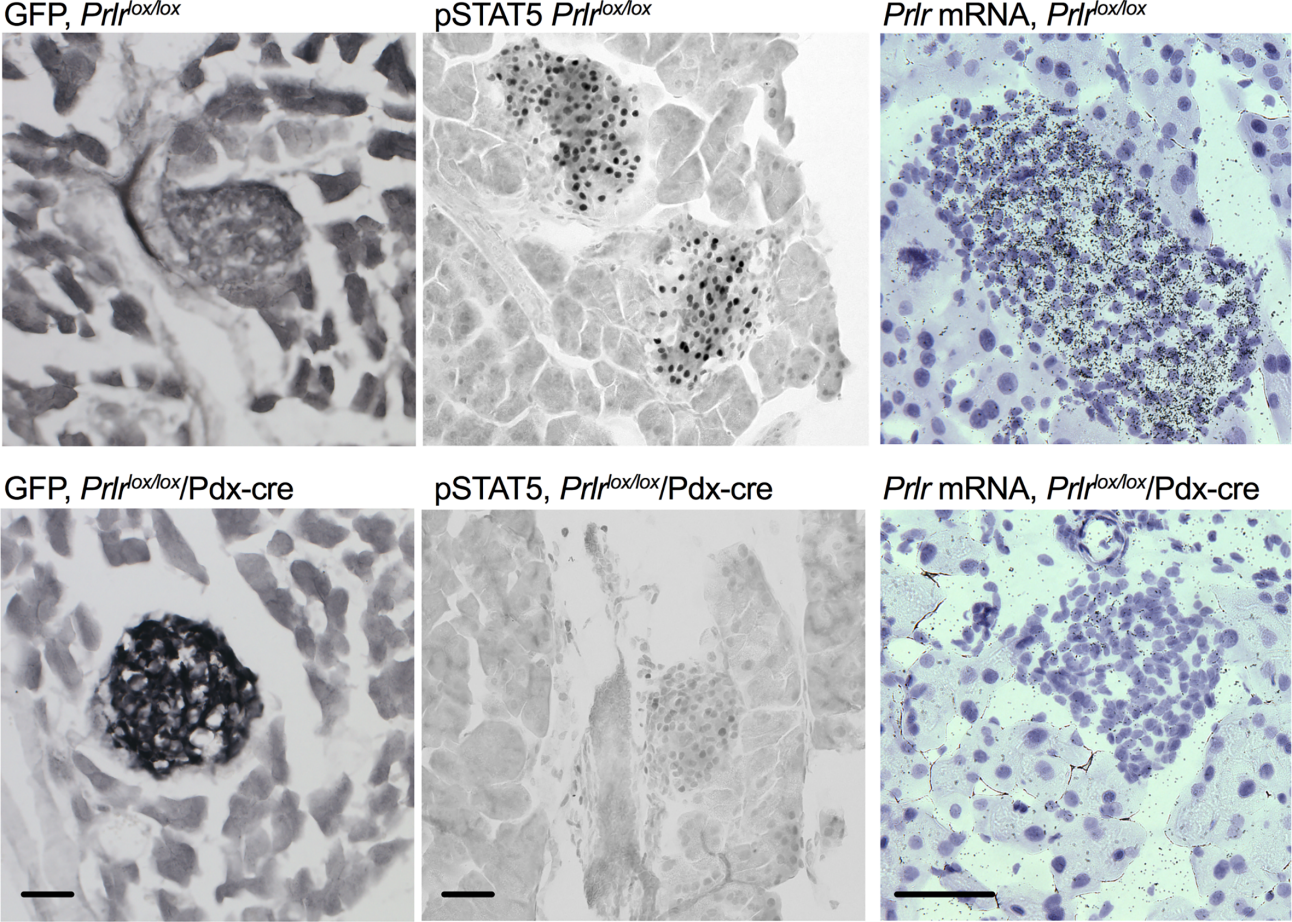
Deletion of Prlr from the pancreas of *Prlr* ^lox/lox^/Pdx-cre mice. Representative images showing GFP immuno-labeling, pSTAT5 immuno-labeling and *in situ* hybridization for the Prlr mRNA in pancreatic tissue from day 15 pregnant *Prlr* ^lox/lox^ (*top*) and *Prlr* ^lox/lox^/Pdx-Cre (*bottom*). Expression of GFP is indicative of the recombination of the targeting construct and deletion of the *Prlr* gene. Recombination-induced GFP expression was only present in the Prlr^lox/lox^/Pdx-Cre mice and was readily observed in pancreatic islets. Pregnant mice have high levels of placental lactogen acting through the Prlr, thus endogenous pSTAT5 can act as a marker of Prlr-containing cells. pSTAT5 was readily detected in islets from *Prlr* ^lox/lox^ pregnant mice but not in *Prlr* ^lox/lox^/Pdx-Cre pregnant mice. *In situ* hybridization images show emulsion-coated sections from the pancreas in which prolactin receptor mRNA is labeled with black silver grains, and nuclei are counterstained purple with Gils hematoxylin. Prlr mRNA was readily detected in islets from *Prlr* ^lox/lox^ mice but was largely deleted in *Prlr* ^lox/lox^/Pdx-Cre mice. Scale bar indicates 50µm.

**Figure 4:**
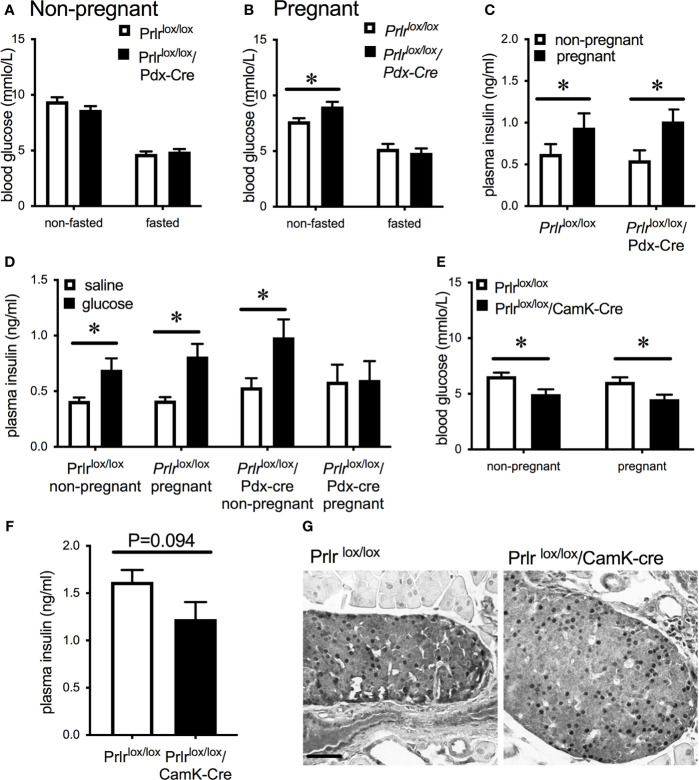
Blood glucose concentrations in non-pregnant **(A)** or day 15/16 pregnant **(B)** *Prlr* ^lox/lox^/Pdx-Cre and control (*Prlr* ^lox/lox^) mice in either the non-fasted or fasted state. **(C)** Plasma insulin concentrations in the non-fasted state in non-pregnant or day 15/16 pregnant *Prlr* ^lox/lox^/Pdx-Cre and control mice. *P < 0.05, Student’s t-test, n = 4 – 10 per group. **(D)** Plasma insulin concentration in non-pregnant and day 15/16 pregnant *Prlr* ^lox/lox^/Pdx-Cre and *Prlr* ^lox/lox^ mice 10 minutes after administration of glucose or vehicle. * = significant effect of glucose treatment, n = 6 – 9 per group. **(E)** Fasting blood glucose concentrations in non-pregnant or day 15/16 pregnant *Prlr* ^lox/lox^/CamK-Cre and control mice (*Prlr* ^lox/lox^). * = significant effect of genotype, n = 12 – 15 per group. **(F)** Non-fasting plasma insulin concentrations from day 16/17 pregnant *Prlr* ^lox/lox^/CamK-Cre and *Prlr* ^lox/lox^ mice (n=7 per group) (Student’s t-test). **(G)** Representative images of pSTAT5 immunohistochemistry in pancreas tissue from day 15/16 *Prlr* ^lox/lox^/CamK-Cre and control (*Prlr* ^lox/lox^) mice showing endogenous pSTAT5 in the islets. Scale bar indicates 50µm.

In the pancreas of Prlr*^lox/lox^*/Pdx-Cre mice, GFP was detected in islets, there was an absence of pSTAT5 during pregnancy in islets, and mRNA for *Prlr* was barely detectable in islets (**Figure 3**), all consistent with a deletion of Prlr in islets in this transgenic mouse. In comparison, islets from control mice showed an absence of GFP, positive Prlr-dependent pSTAT5 signaling during pregnancy (in response to endogenous placental lactogen) and readily detectable mRNA for the *Prlr* (**Figure 3**). Blood glucose levels were similar in non-pregnant *Prlr* ^lox/lox^/Pdx-Cre and control mice in both the fasted and fed state (**Figure 4A**). Pregnant *Prlr* ^lox/lox^/Pdx-Cre had elevated blood glucose levels in the fed state compared to pregnant control mice (Student’s t-test P=0.0358), while fasted blood glucose levels were similar (**Figure 4B**). Plasma insulin concentrations in the fed state did not differ between the genotypes, but there was an increase in insulin concentrations in pregnancy compared to the non-pregnant state for both the *Prlr* ^lox/lox^/Pdx-Cre and control mice (two-way ANOVA, effect of physiological state P=0.0228, F(1,16)=6.341) (**Figure 4C**). GSIS was impaired in pregnant *Prlr* ^lox/lox^/Pdx-Cre (two-way ANOVA, interaction between treatment and physiological state P=0.0295, F(1,25)=5.332) (**Figure 4D**). In both the non-pregnant and pregnant state, *Prlr* ^lox/lox^/CamK-Cre had significantly lower fasting blood glucose than control (*Prlr* ^lox/lox^) mice (two-way ANOVA, effect of genotype P=0.0003 F(1,49)=15.27) (**Figure 4E**). Non-fasting insulin concentrations were similar in pregnant *Prlr* ^lox/lox^/CamK-Cre and controls (**Figure 4F**). Immunoreactivity for pSTAT5 was readily detectable in the islets of pregnant *Prlr* ^lox/lox^/CamK-Cre and *Prlr* ^lox/lox^ mice (**Figure 4G**) supporting no off-target deletion of Prlr in the pancreas in *Prlr* ^lox/lox^/CamK-Cre mice.

**Figure 5:**
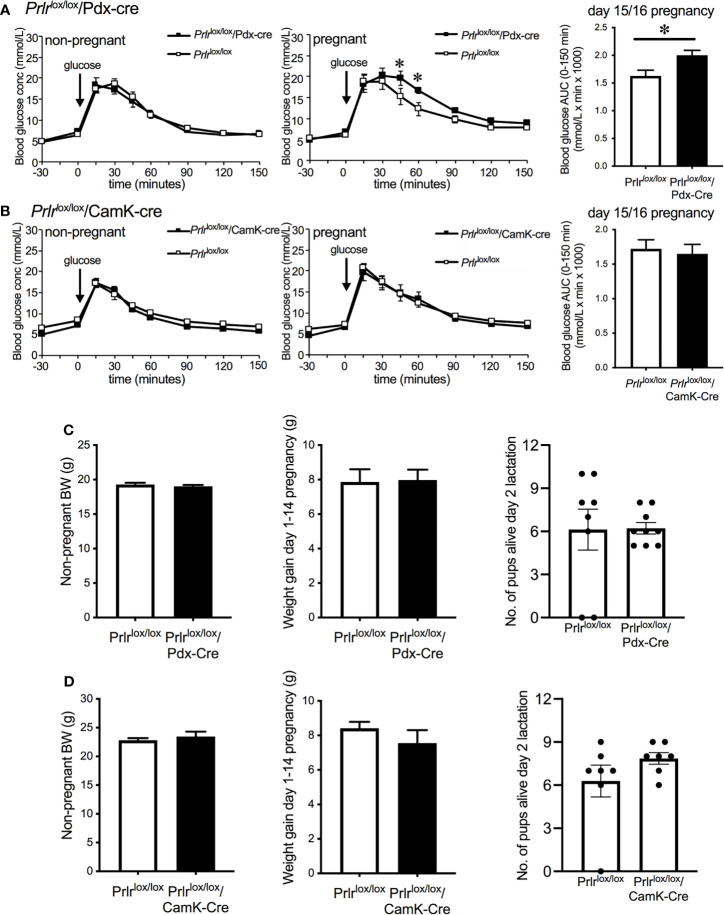
Glucose tolerance in non-pregnant and day 15/16 pregnant *Prlr* ^lox/lox^/Pdx-Cre **(A)**, *Prlr* ^lox/lox^/CamK-Cre **(B)** and control **(A, B)** mice. Blood glucose was monitored before and after the administration of 1g/kg glucose in fasted mice and bar graphs show area under the curve (mean ± SEM) during glucose tolerance tests in pregnant groups, * = significantly different from controls, n = 8 – 15 per group. Non-pregnant body weight, weight gain from day 1-14 of pregnancy and litter size on day 2 of lactation for *Prlr* ^lox/lox^/Pdx-Cre **(C)** and *Prlr* ^lox/lox^/CamK-Cre **(D)**.

Non-pregnant *Prlr* ^lox/lox^/Pdx-Cre and *Prlr* ^lox/lox^/CamK-Cre mice had similar glucose tolerance to *Prlr* ^lox/lox^ control mice (**Figures 5A, B**). Pregnant *Prlr* ^lox/lox^/Pdx-cre mice had significantly impaired glucose tolerance compared to control pregnant mice (Student’s t test, P=0.0166) (**Figure 5A**). Using the *Prlr* ^lox/lox^/CamK-Cre mice to control for possible effects of prolactin action in the brain, our results show there was no different in glucose tolerance between *Prlr* ^lox/lox^/CamK-Cre and control mice in either the non-pregnant or pregnant state (**Figure 5B**). Non-pregnant body weight, gestational weight gain from day 1 to day 14 (prior to fasting for GTT) and number of live pups per litter did not differ between *Prlr* ^lox/lox^/Pdx-Cre (**Figure 5C**) and *Prlr* ^lox/lox^/CamK-Cre (**Figure 5D**) and their respective controls.

**Figure 6:**
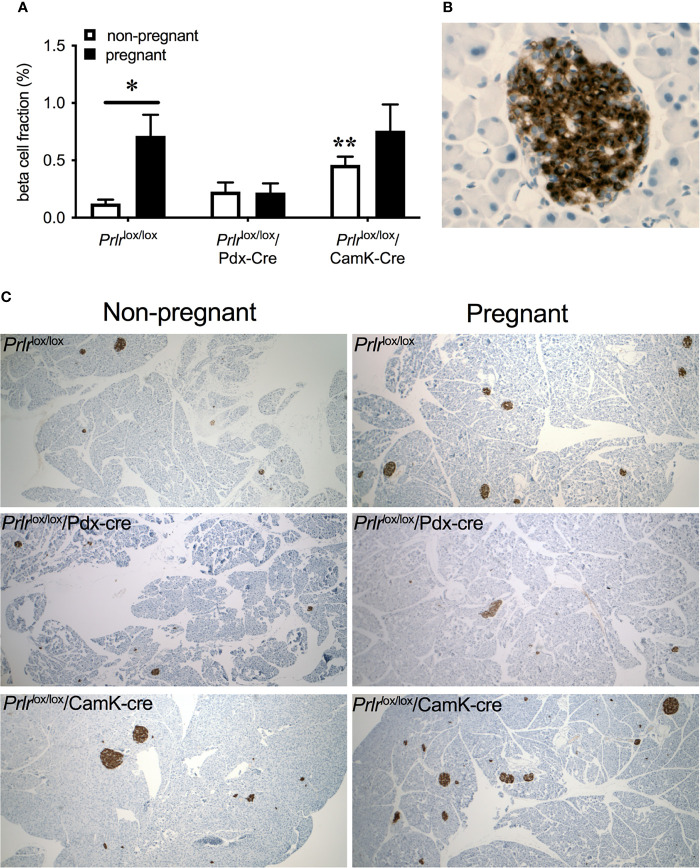
Beta-cell fraction of pancreas (mean± SEM) from non-pregnant and pregnant control (*Prlr* ^lox/lox^), *Prlr* ^lox/lox^/Pdx-Cre and *Prlr* ^lox/lox^/CamK-Cre mice **(A)**. * = significantly differently from virgin group of same genotype, ** virgin group significantly different to non-pregnant *Prlr* ^lox/lox^ group, n = 5 – 8 per group. High magnification image **(B)** illustrating islet cells positively immuno-stained for insulin. Representative images **(C)** of pancreatic tissue immuno-stained for insulin from each group.

In control mice, pregnancy led to a significant increase in beta-cell fraction of the pancreas (two-way ANOVA, interaction physiological state X treatment P=0.0133, F(1,20)=7.373, Sidak’s multiple comparison test control non-pregnant vs pregnant P=0.0022) (**Figure 6**). This increase in beta-cell fraction was not observed in the pregnant *Prlr* ^lox/lox^/Pdx-Cre mice (Sidak’s multiple comparison test *Prlr* ^lox/lox^/Pdx-Cre non-pregnant vs pregnant P=0.998) nor *Prlr* ^lox/lox^/CamK-Cre (Sidak’s multiple comparison test *Prlr* ^lox/lox^/CamK-Cre non-pregnant vs pregnant P=0.438), suggesting that this effect was driven by prolactin action directly on the beta cells (**Figure 6**). Pregnant *Prlr* ^lox/lox^/CamK-Cre mice had similar beta-cell fraction as pregnant control mice (Sidak’s multiple comparison test P=0.5727), confirming that this action of prolactin was not mediated by Prlr in the forebrain (**Figure 6**).

## Discussion

Increasing concentrations of prolactin/placental lactogen play a key role in the adaptations of the beta-cell to pregnancy (1, 2). Identification of the site of action is an important step to determining the mechanism of prolactin receptor activity, and if this will be a useful target for intervention. Our work has replicated previous studies that have deleted Prlr specifically from the beta-cells (11), pancreas (12), and adult onset deletion from the pancreas (42) demonstrating that Prlr activity in the pancreas is required for maternal adaptations in beta-cells during pregnancy. Moreover, in the current study we have demonstrated that these transgenic mice lines with beta-cell- or pancreas-specific deletions of the Prlr also show deletion of Prlr in the hypothalamus, particularly the arcuate nucleus, an area known to contribute to central regulation of insulin secretion and glucose homeostasis (18–20, 35, 43, 44). This hypothalamic deletion of Prlr is not unexpected as many transgenic lines that express Cre in the pancreas also express Cre in the brain (13–15) and previously we have shown that at least one of these lines induced deletion of the Prlr in several nuclei known to be involved in whole body glucose homeostasis (17). Using a mouse with a forebrain specific deletion of Prlr, we have shown that there is no impaired beta-cell expansion, glucose tolerance or insulin concentration during pregnancy in the absence of Prlr from the forebrain, including in the arcuate nucleus. These results confirm that the beta-cell is the direct site of action for prolactin and placental lactogen mediated changes in beta-cell adaptation during pregnancy and is independent of actions of prolactin and placental lactogen in the forebrain.

Firstly, using the *Prlr* ^lox/lox^ */*Rip-Cre mice, our data replicated earlier findings that showed impaired glucose regulation during pregnancy in mice with deletion of Prlr from beta-cells (11), but there were several factors which made the interpretation complicated. In the current study, the Rip-Cre^Tg25Mgn^ line showed that Cre by itself had an effect on glucose tolerance during pregnancy. This transgene effect has been observed in some studies with this Rip-cre line, but not all (45). Interestingly, while the effect of the Rip-Cre transgene may be masking any potential specific effects of Prlr on glucose tolerance during pregnancy, elevated non-fasting blood glucose, an indicator of maladapted glucose regulation during pregnancy, was only detected in the *Prlr^lox/lox^/*Rip-Cre mice and not the control Rip-Cre mice, suggesting a specific role for the Prlr in the pancreas in agreement with the previously published pregnant Rip-Cre^Herr^ Prlr KO mouse (11). The Rip-Cre^Tg25Mgn^ transgenic line is thought to be more susceptible to the Cre transgene effect (46), and this likely explains the differences in results between the Cre-only controls for each line.

We also used the Pdx-cre to delete Prlr from the pancreas and showed pregnancy-specific impaired adaptions in glucose regulation, as demonstrated by impaired glucose tolerance, suppressed GSIS, higher non-fasting blood glucose levels and impaired beta-cell expansion, similar to previous work (12). The Pdx-cre line has Cre recombinase expressed more widely in the pancreas than the Rip-cre line, and therefore is not specific to just the beta-cells within the pancreas, and there is also some expression in the duodenum and hypothalamus (14, 15, 47). In the pancreas in adults, Prlr is most intensely expressed in islets, with serial immunohistochemistry suggesting that expression is mostly on beta-cells, not alpha-cells (48). During early development, Prlr immuno-reactivity is primarily in acinar cells and ducts, then, later in development, expression is mostly in pancreatic islets (49). Thus, it is likely that in the *Prlr* ^lox/lox^/Pdx-Cre mice, Prlr is deleted from other pancreatic cell types along with beta-cells. Due to the design of our *Prlr* ^flox^ construct, deletion of Prlr results in expression of GFP under the control of the Prlr promoter (36). Our observation of GFP in the pancreas of *Prlr* ^lox/lox^/Pdx-Cre mice indicates that the islets are the predominant location of the deletion of Prlr in the pancreas.

A critical omission from previous studies examining the effect of specific deletion of Prlr from the pancreas (11, 12) was an examination of the brain to determine the extent to which central Prlr were affected in these transgenic mice lines (15). This has been somewhat addressed in a recent study in mice in which Prlr was deleted from the pancreas in adulthood using a tamoxifen-inducible Cre line, as mRNA for Prlr in the entire hypothalamus was reported to be no different to control mice (42). Given that we only detected recombination of the Prlr gene in a small population of cells in the arcuate nucleus using the Pdx-cre line, however, and that Prlr is extensively expressed in the hypothalamus (16) it is perhaps not surprising that no change of Prlr mRNA would be detected when assessing the entire hypothalamus as a whole. Thus, a role of brain Prlr activation in contributing to pregnancy-induced changes in glucose homeostasis still cannot be ruled out. The Rip-Cre^Herr^ line has less extensive expression of Cre in the brain compared to the Rip-Cre^Tg25Mgn^ line but Cre is still present, particularly in metabolic-sensing neurons in the arcuate nucleus (15, 50).The Pdx-cre line has even more limited Cre recombinase expression in the brain, yet this is also mostly found in the medial basal hypothalamus, a region containing the arcuate nucleus (14, 15, 47) and a high number of Prlr expressing cells (16, 41). Our current results, along with our previously published observations (17), demonstrate that both *Prlr^lox/lox^/*Rip-Cre and *Prlr* ^lox/lox^/Pdx-Cre had extensive deletion of Prlr in the arcuate nucleus. To determine possible involvement of the central Prlr in the effects of prolactin/placental lactogen on glucose homeostasis during pregnancy, mice with specific deletions of forebrain Prlr, *Prlr* ^lox/lox^/CamK-Cre mice, were included in the current study. The mice with specific deletion of Prlr in forebrain neurons, with its much more extensive deletion of Prlr from the arcuate nucleus and other brain regions (36), should fully account for any possible effect of the loss of Prlr in the arcuate nucleus in *Prlr* ^lox/lox^/Pdx-Cre and *Prlr* ^lox/lox^/Rip-Cre mice. During pregnancy, glucose tolerance was similar in mice lacking Prlr in forebrain neurons and controls, and similar beta-cell fraction was observed in pregnant *Prlr* ^lox/lox^/CamK-Cre as pregnant control mice, indicating that despite the lack of central Prlr (36, 41) beta-cell expansion during pregnancy remained unaffected. Thus, these data support the role of a pancreas-specific effect of Prlr activation in the pregnancy-induced adaptations of the beta-cells with no major influence of central Prlr.

Interestingly, while glucose tolerance in *Prlr* ^lox/lox^/CamK-Cre mice was unaffected in either the non-pregnant or pregnant state, the non-pregnant *Prlr* ^lox/lox^/CamK-Cre mice had significantly increased beta-cell fraction compared to non-pregnant control *(Prlr* ^lox/lox^) mice. Non-pregnant *Prlr* ^lox/lox^/CamK-Cre mice are hyperprolactinemic since the removal of Prlr from tuberoinfundibular dopamine neurons in the arcuate nucleus leads to impaired negative feedback of prolactin secretion (36). Thus, it seems likely that the non-pregnant *Prlr* ^lox/lox^/CamK-Cre mice are already exhibiting a pregnancy-like beta-cell expansion due to chronic high prolactin concentrations, as has previously been shown *in vitro* (51). These data further highlight the direct role of prolactin/placental lactogen in the pancreas and a lack of requirement of central Prlr to induce beta-cell expansion. Lower fasting glucose concentrations in both the non-pregnant and pregnant state were also observed in the mice with the forebrain deletion of Prlr. The mechanism underlying this is yet to be determined but it may also be due to the chronically elevated prolactin concentrations impacting on glucose regulation in the periphery. In human studies, elevated prolactin concentrations can be protective against the development of type 2 diabetes and impaired glucose regulation, yet also can be associated with insulin resistance and hyperglycemia [reviewed in (52, 53)]. The interaction of prolactin and peripheral glucose regulation is clearly complex, and going forward it may be that the hyperprolactinemic *Prlr* ^lox/lox^/CamK-Cre, with its low fasting blood glucose, will be a useful model to further our understanding of this interaction.

Most of the work investigating prolactin/placental lactogen-induced beta-cell expansion during pregnancy has been done in rodent models, and caution needs to be applied before inferring that these mechanism will be the same in humans [reviewed in (1)], not least because the placental complement of hormones is quite different (54, 55). There is only limited evidence of beta-cell expansion during human pregnancies [reviewed in (1, 56)], and this evidence suggests it is markedly less extensive than that seen in rodent models and potentially involves neogenesis of beta cells rather than cell division of existing beta cells (57). Studies examining prolactin during human pregnancy have not consistently found abnormal values associated with gestational diabetes (58–60), but such studies often do not account for both prolactin and placental lactogen together - clearly absolutely necessary in terms of ligand-induced activation of the Prlr during pregnancy. In addition, there is controversy over whether human beta-cells actually express Prlr. While some *in vitro* studies have shown proliferation of human islets cells in response to lactogenic hormones (61) others have not (62). These inconsistent data may be explained by evidence from non-pregnant human tissue suggesting minimal expression of Prlr on beta-cells (63). Further work in tissue from pregnancy is required, however, because Prlr expression may be regulated at this time. In mice, protein levels of Prlr in the pancreas are barely detectable in virgin mice, but increase in pregnancy (11). Transcriptomic data suggest that the Prlr is expressed in human beta cells, although at lower levels than seen in rodents (64). Again, an important caveat is that transcriptome data for humans came from post mortem subjects (both male and female), mostly beyond reproductive age. There is also evidence from mice that non-beta cells in the pancreas are important for Prlr related effects on beta-cells (42) and Prlr expression is also enriched in non-beta cells from the human pancreas (64). In mice, alpha-cells in the pancreas undergo Prlr-induced adaptations in function during pregnancy (65). Thus, much work is still required to evaluate the full mechanism of Prlr action on glucose homeostasis in human pregnancy. Prlr gene polymorphisms have been associated with the development of gestational diabetes in humans (66) suggesting that despite uncertainty over the mechanisms, changes in lactogenic signaling are likely to be important in adaptive change in glucose homeostasis in women during pregnancy, as it clearly is in rodents.

Overall, the data here confirm a direct action of prolactin/placental lactogen in the pancreas in glucose regulation during pregnancy in mice. Key new data provided by the present study is the evidence that deletion of Prlr in the hypothalamus does not appear to contribute to the adaptive changes in glucose homeostasis in pregnancy. Prior data are not adequately interpretable without this information (11, 12, 15). Moreover, we have shown that hyperprolactinemia caused by absence of Prlr in the forebrain also induces beta-cell expansion and “pregnancy-like” changes in glucose homeostasis, further evidence for a direct action of elevated prolactin in the pancreas. During pregnancy, mice with specific deletion of Prlr from the pancreas display impaired beta-cell expansion, attenuated GSIS and impaired glucose tolerance demonstrating a key role for pancreatic Prlr during pregnancy. Although both “pancreas-specific” Cre lines caused some recombination in the hypothalamus, this is unlikely to have had an effect on glucose homeostasis, as global deletion of Prlr in the forebrain did not alter these normal pregnancy-induced changes. There are a range of pancreas specific cre-lines available, and in the current study we only used two different transgenic mouse Cre lines to target Prlr removal from the pancreas. These transgenic lines used were similar to what has previously been published investigating the role of prolactin receptor activation in maternal adaptations of the beta-cell (11, 12). Currently, no beta-cell or pancreas specific cre line is without complications (13, 15, 46, 67, 68) and even AAV8 Ins1-cre viral injection into the pancreatic duct can have off target effects (69). Our replication of previous work, along with the novel assessment of the forebrain deletion of Prlr to investigate the contribute of prolactin action in the brain, strongly supports the conclusion that this particular adaptive response is mediated in the periphery, independent of central actions of prolactin.

## Data Availability Statement

The raw data supporting the conclusions of this article will be made available by the authors, without undue reservation.

## Ethics Statement

The animal study was reviewed and approved by University of Otago Animal Ethics Committee.

## Author Contributions

SL and DG. designed the research. ZA, SL, and IK performed research. ZA and SL analyzed data. SL, ZA, and DG wrote the paper. All authors contributed to the article and approved the submitted version.

## Funding

Health Research Council of New Zealand (Program Grant 14-568) and Maurice Wilkins Centre for Molecular Biodiscovery provided grant funding to cover salaries and working expenses for this project.

## Conflict of Interest

The authors declare that the research was conducted in the absence of any commercial or financial relationships that could be construed as a potential conflict of interest.

## Publisher’s Note

All claims expressed in this article are solely those of the authors and do not necessarily represent those of their affiliated organizations, or those of the publisher, the editors and the reviewers. Any product that may be evaluated in this article, or claim that may be made by its manufacturer, is not guaranteed or endorsed by the publisher.
